# A case of giant inguinoscrotal hernia managed by preoperative pneumoperitoneum with an unforeseen complication and outcome: a case report and review of literature

**DOI:** 10.1007/s10029-023-02870-4

**Published:** 2023-09-06

**Authors:** A. H. El Shamarka, M. H. Zidan, M. S. Youssef, A. H. El Banna, M. Mourad

**Affiliations:** 1 General Surgery Department, Main University Hospital, Alexandria University, Alexandria, 21568 Egypt; 2 General Surgery Department, Alexandria Medical Research Institute Hospital, Alexandria University, Alexandria, Egypt; 3 Faculty of Medicine, Alexandria University, Alexandria, 21568 Egypt

Mega hernias or giant inguinoscrotal hernias (GISH) are peculiar presentations of inguinoscrotal hernias, that can attain extraordinary proportions. GISHs are defined as hernias that extend below the midpoint of the inner thigh in the standing position [1]. These hernias are distinguished by a substantial and massive hernial sac, causing significant morbidity, and posing unique diagnostic and management challenges.

GISHs are often associated with chronicity and neglect, with patients presenting late due to various factors including fear, limited access to healthcare, and socioeconomic disparities. The considerable size of the hernia can result in compression of adjacent structures, leading to symptoms such as pain, discomfort, and impaired mobility. These massive hernias pose significant problems, as the replacement of the herniated viscera causes an abrupt rise in the intra-abdominal pressure, which compromises the cardiorespiratory system [2]. In addition to the typical hernia symptoms, these patients frequently experience trouble voiding, urine retention, and the possibility of pressure ulcers along the lateral area of the scrotum, infection, and decreased mobility. Moreover, the penis is buried inside the scrotum, permitting urine to drip into the already thin and delicate skin of the scrotum, which is swollen from lymphatic and venous edema, leading to excoriation, ulceration, and secondary infection, these issues have a significant psychological impact and cause social isolation [3]. Therefore, the management of giant inguinoscrotal hernias necessitates meticulous evaluation, surgical expertise, and a multidisciplinary approach involving general surgeons, urologists, interventional radiologists, and/or plastic surgeons.

Despite advancements in surgical techniques and technology, the management of giant inguinoscrotal hernias remains complex, and the literature on this topic is limited. Therefore, the objective of this case report is to present a challenging case of a GISH and provide a comprehensive review of the current literature on the diagnosis, evaluation, and management of this rare condition. By elucidating the clinical features, diagnostic considerations, and treatment modalities, this paper aims to contribute to the existing scientific knowledge and enhance the understanding of this complex condition among healthcare professionals.

We are presenting a case of a 43-year-old male patient, who presented to the general surgery clinic with a large inguinal hernia that has been present since childhood, the patient had no significant medical or surgical history. His symptoms included bouts of constipation, skin irritation, and difficulty in micturition, these symptoms progressed in severity and frequency over time until he sought medical advice.

Physical examination revealed a huge right-sided inguinoscrotal hernia extending to mid-thigh, Type II as per Trakarnsagna classification [4]. Computerized tomography (CT) scan of the abdomen and pelvis demonstrated a large hernial sac containing the caecum, ascending colon, transverse colon, ileal bowel loops, and marked fluid collection.

The patient was admitted to Alexandria Main University Hospital and was planned for artificial pneumo-peritoneum to facilitate reduction of the content and avoid postoperative abdominal compartmental syndrome. A target volume of 10 L of air gradually introduced over the course of 20 to 25 days was deemed sufficient for the hernia’s safe reduction. A 12 French pigtail catheter was inserted intra-abdominally by the intervention radiologist and we proceeded with inflation of the abdomen with 400–500 cc of air daily depending on the patient’s tolerance using a sterile syringe and manual daily bedside inflation (Figs. 1, 2 and 3).

**Fig. 1:**
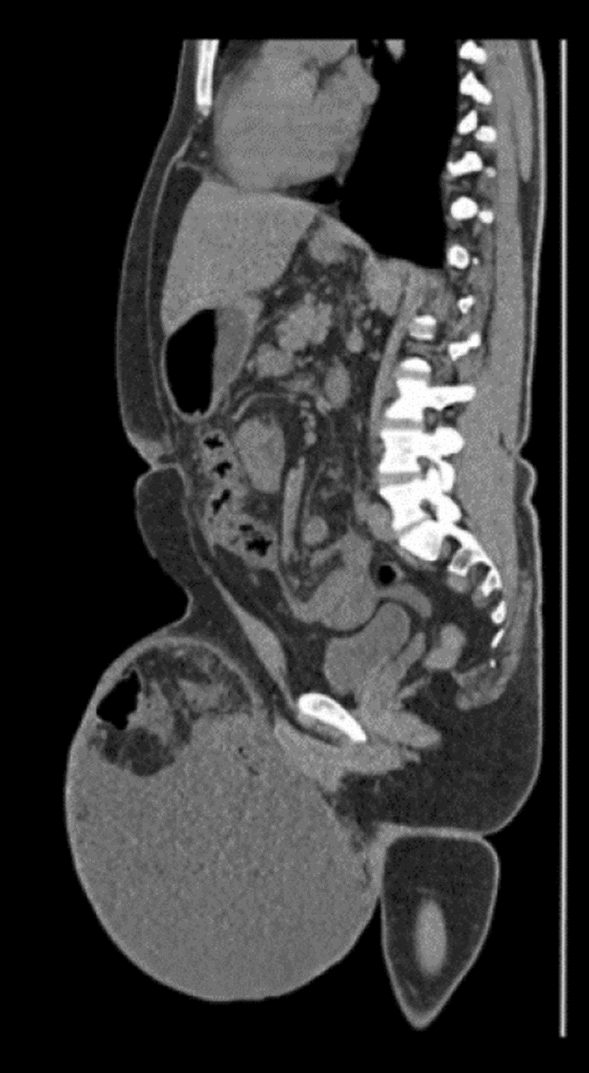
CT scan of the patient’s abdomen before PPP

**Fig. 2:**
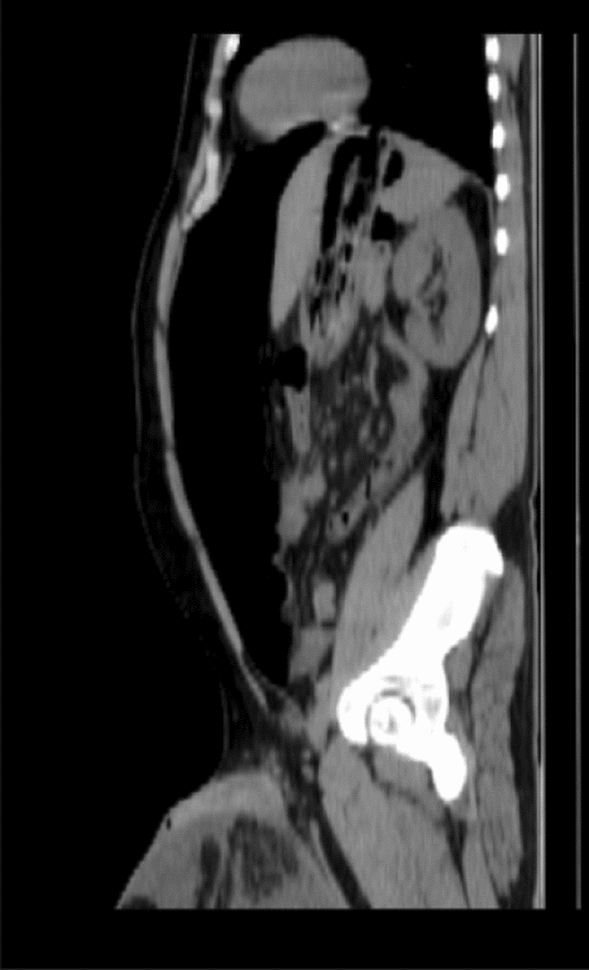
CT scan of the patient’s abdomen after PPP

**Fig. 3:**
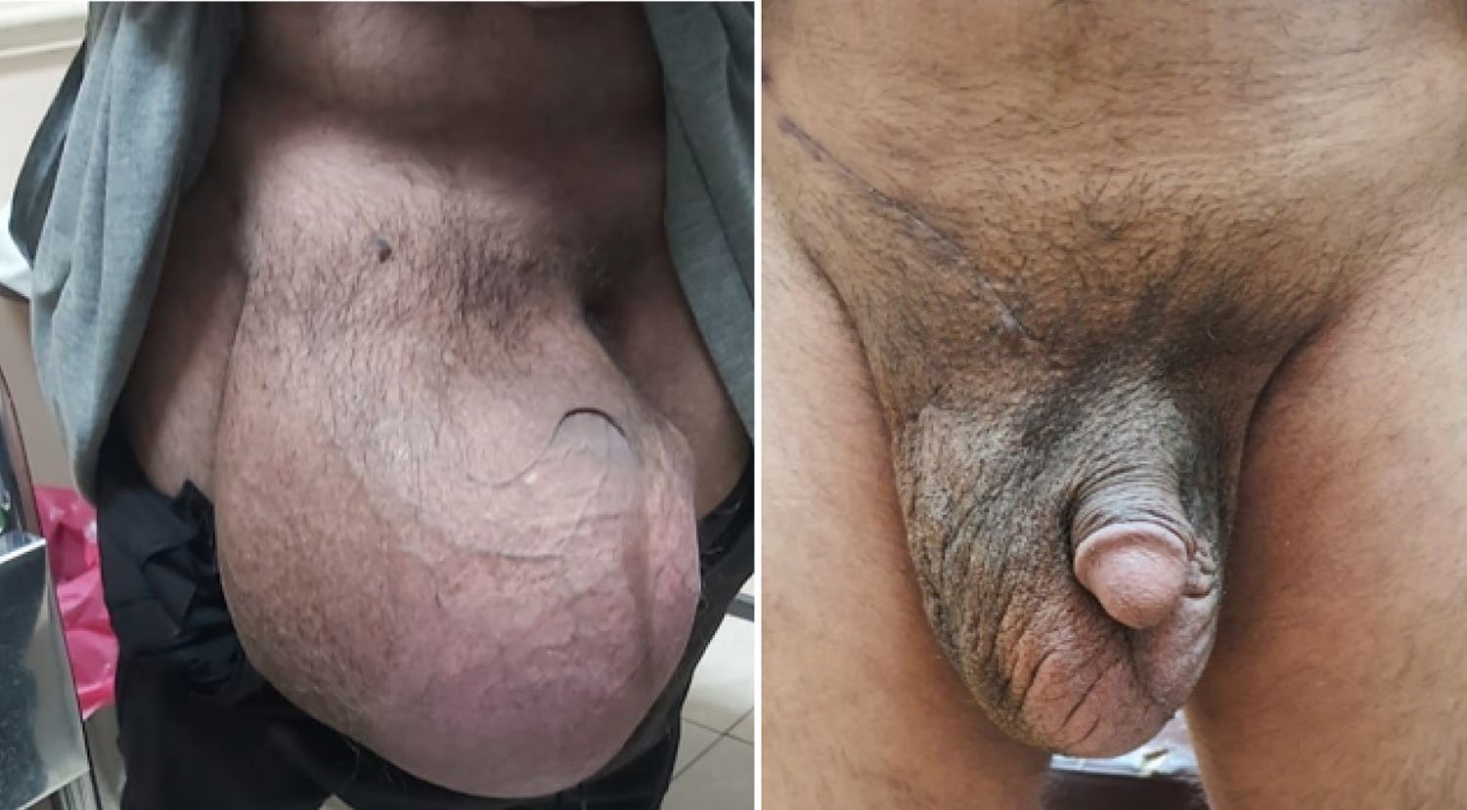
Preoperative photo of the patient’s hernia compared to post-operative photo

Serial CT scans of the abdomen were used to assess the pneumoperitoneum’s progression and ensure the intra-abdominal volume was sufficient for the complete reduction of the hernia. With the gradual introduction of air into the peritoneal cavity the patient developed progressing dyspnea with every session. The maximum volume that was tolerated by the patient in the provided timeline was 7 L. The decision was made to proceed with hernioplasty; however, on the morning of the surgery, the patient refused the surgery and refused to remove the pigtail and left the hospital in hopes to get re-admitted in the following days for surgery. After two weeks, the patient returned to the hospital with abdominal pain, fever, vomiting, and signs of peritonitis. Laboratory investigations revealed a white count of 4.8 × 10^3^/uL and CRP 202, indicating early sepsis. ulc.

A follow-up CT scan of the abdomen revealed moderate collection all over the abdomen and multiple loculated collections, the largest at the sub-hepatic region and the pelvis, The patient’s pigtail which was seen ending in the collection, was opened into a retrieval collection bag, the bag was immediately filled with 1 L of pus. Cultures were drawn from the collection for culture and sensitivity and serial drainage of the loculated collections was performed by interventional radiologists and another pigtail was inserted in the pelvic collection.

Daily assessment of the drain output was performed in addition to serial ultrasound assessment of the intra-abdominal collection in combination with culture-based antibiotics used for the treatment of the intra-abdominal infection. Over a period of 8 weeks, the abdominal drains’ output showed marked regression, and the drains were removed, leaving only the pelvic drain.

A follow-up CT scan showed near total resolution of the intra-abdominal collections; however, marked regression of the artificial pneumoperitoneum, and progression of a moderate scrotal collection were noted compared to the first CT scan on the patient. The progression of the scrotal collection led to the reduction of the hernial sac contents, and as such a final drain was placed in the scrotal collection, and a 3–4 L of a turbid serous scrotal aspirate was drained initially. Over the following two weeks, there was a regression of the drain output, and a serial ultrasound was done until total regression of the scrotal collection, and the patient was rescheduled for hernioplasty.

An inguinal incision was made, along the whole length of the inguinal ligament, and the opening of the inguinal canal revealed a large sac with its contents partially reduced. Reduction of the sac was undergone, and a large previously drained abscess cavity was seen adherent to the cord. Dissection of the abscess cavity was done, with complete excision. There was no spillage or pus content inside the cavity; therefore, Liechtenstein tension-free mesh repair was done, and a negative suction drain was inserted before the closure of the wound. Scrotal skin excision has its complications and thus the redundant scrotum was left for conservative treatment.

The postoperative course was unremarkable, the patient was mobile on the same day of the operation, oral feeding was started the following day with no signs of obstruction or increased intra-abdominal pressure, no tachypnea or decrease in the patient’s oxygen saturation was noted, also measurement of urine output and renal function tests was performed daily and where within normal range, the drain showed less than 100 ccs of serous fluid daily and the patient had mild scrotal skin edema. The patient was discharged 5 days postoperatively with the drain still in place and instructions on wound care and daily drain evacuation and assessment of its output.

The patient was on a weekly follow-up schedule for the first two weeks postoperatively. In the first week, there was a marked regression of the redundant scrotal skin with residual scrotal skin edema, and the drain output was below 50 ccs of serous fluid daily. The drain was removed in the second post-operative week. No wound-related complications were noted in the follow-up. The one-month follow-up was unremarkable with the patient having improved mobility and dramatic improvement in daily activities where previously hindered by the hernia, no skin irritation or infections were noted with an overall improvement in the patient’s quality of life.

Reduction and repair of a GISH is a challenging surgical procedure and is linked with substantial morbidity and mortality, resulting from returning the herniated organs to the empty abdominal cavity [5]. A giant inguinal hernia is associated with psychological and social impact due to the patient’s discomfort, difficult mobilization, non-fitting of clothes, and sexual discomfort.

The terms “loss of domain (LOD)”, or “loss of abdominal domain”, have recently been widely used in literature to describe the distribution of abdominal content between the hernia and residual abdominopelvic cavity [6]. A recently written definition consensus to the term has been proposed. LOD defines large hernias with difficult irreducibility due to lack of intra-abdominal space, which entails reconstructive surgery that would increase the risk of complications due to raised intra-abdominal pressure [7]. Intraabdominal hypertension (IAH) or abdominal compartment syndrome (ACS) could develop due to the disproportion of the abdominal cavity domain and the large content of the reduced hernial sac, posing an increased risk of morbidity and mortality in the peri-operative period [8]. Furthermore, reduction of the hernial content would affect the integrity, and elasticity of the abdominal wall muscles, resulting in possible atrophy and fibrosis of the abdominal wall; thus, increasing the risk of IAH [9].

Although there is not yet a consensus regarding the classification and management of GISH; Trakarnsagna proposed a simple grading system to describe GISH [4]. Trakarnsagna classification has not yet been applied to a systematic study, nor has it been tested in literature to carefully depict GISH. Trakarnsagna proposed management options for his grades; however, there are no current guidelines, nor systematic reviews that recommend a specific management modality for any of his grades.

Many management options have been proposed throughout the literature, but none have been carefully studied as to which would be the best options. All the management options proposed were through case reports or series, and no comparative studies have yet been conducted.

Surgical reduction of GISH without preoperative interventions such as the “hug” technique presented by Cavalli in 2015 [10], has not been tested in clinical practice. The risk of postoperative cardiorespiratory compromise and IAH should still be taken into consideration.

Surgical resection or debulking of the hernial contents is an option for treatment to decrease intra-abdominal pressure. However, debulking surgeries may entail bowel resection, omentectomy, and/or colectomy with or without covering ileostomies. Resection and anastomosis are associated with the risk of anastomotic leakage, and infection of the prosthesis from resected bowel. Furthermore, the need for ileostomies would entail further operations. Thus, resection of the hernial content might be associated with high morbidity rates, in terms of ileostomies, leakage, post-reduction cardiopulmonary compromise, and post-operative need for intensive care monitoring and re-operation [4, 11, 12]. More studies are still needed to re-evaluate debulking surgeries and their morbidity rates.

Enlarging the abdominal cavity by major procedures such as phrenectomy, creation of a ventral hernia using mesh and scrotal skin or dartos muscle flaps, and abdominal wall component separation techniques have been described in the literature in efforts to decrease the post-operative risk of increased intra-abdominal pressure [12]; however, the usage of such techniques may be associated with high morbidity. These major operations can be replaced by less invasive techniques, such as botulinum toxin injection, and progressive pneumoperitoneum instillation.

Botulinum toxin A (BTX) injection in the abdominal wall has been described as temporary muscle paralysis without systemic effects. Injection of BTX, for 6 to 45 days (about 1 and a half months), into the muscle can paralyze and elongate the abdominal muscles: thus, increasing the abdominal cavity space [13]. However, BTX injection is associated with a prolonged hospital stay, and high cost [14].

Moreno first described the use of preoperative progressive pneumoperitoneum (PPP) therapy in ventral hernias [15], and many authors have proposed its usage in GISH as a safe modality to prevent post-operative IAH or ACS [4, 16, 17]. Preoperative progressive pneumoperitoneum (PPP) is a technique that has been used in the management of giant inguinoscrotal hernias. This technique involves daily gradual insufflation of 500 cc–2000 cc of carbon dioxide gas into the peritoneal cavity for 7–14 days (about 2 weeks) to increase the intra-abdominal pressure and increase the size of the hernial sac [18]. The gradual increase in intra-abdominal pressure also helps to improve respiratory function and reduce the risk of cardiorespiratory peri-operative complications. However, the usage of PPP is associated with long hospital stays and induction of infection. Overall, PPP is a valuable technique that can improve outcomes in the management of GISH [17].

GISH repair is associated with postoperative scrotal skin redundancy, especially when managed by PPP, owing to the reduction of content, and increasing the abdominal domain; thus, replacing the scrotal space with peritoneal collections, with the risk of abscess formation, if hernioplasty was delayed. The considerable drainage of scrotal collections might be studied carefully, and its incidence should be evaluated in GISH, and after the usage of PPP [19, 20].

Postoperative scrotal skin redundancy can be left for follow-up, as scrotal skin elasticity carries marvelous results to retain its normal size; however, postoperative scrotal hematomas can be evident after the reduction of long-standing hernias. Formation of neo-scrotum has also been suggested in the literature, with or without the usage of flaps [21, 22]; nonetheless, there are no studies that compare both techniques regarding the postoperative results and complications.

Studies have shown that PPP can effectively increase intra-abdominal pressure. PPP was associated with facilitating the reduction of the hernial content and decreased rate of complications in patients with GISH [17, 23] . Many case reports provide evidence to support the use of PPP as a safe and effective technique in the management of GISH; However, more studies and systematic reviews are needed to evaluate the usage of PPP and compare PPP with other modalities currently used to avoid post-operative IAH in the management of GISH such as BTX injection in respect to complication rates, hospital stay, and cost-effectiveness.

In conclusion, GISH is a rare presentation of all inguinoscrotal hernias, that is managed by various lines, as there still is no consensus or a standard guideline. Current evidence of management depends solely on a case-by-case basis, and there is still no evidence to support which line is better in what circumstances. A consensus should be reached to clearly define, grade, and manage GISH.

## Data Availability

All data generated or analyzed during this study are included in this published article.

## Abbreviations

GISH: Giant inguinoscrotal hernia
CT: Computerized tomography
BTX: Botulinum toxin A
IAH: Intra-abdominal hypertension
ACS: Abdominal compartment syndrome
CRP: C-reactive protein
PPP: Progressive preoperative pneumoperitoneum
